# Effectiveness of ozone-laser photodynamic combination therapy for healing wounds infected with methicillin-resistant *Staphylococcus aureus* in mice

**DOI:** 10.14202/vetworld.2023.1176-1184

**Published:** 2023-05-31

**Authors:** Suryani Dyah Astuti, Wahyu Intan Pertiwi, Sri Puji Astuti Wahyuningsih, Perwira Annissa Dyah Permatasari, Dezy Zahrotul Istiqomah Nurdin, Ardiansyah Syahrom

**Affiliations:** 1 Department of Physics, Faculty of Science and Technology, Airlangga University, Surabaya, 60115, Indonesia; 2 Department of Biology, Faculty of Science and Technology, Airlangga University, Surabaya, 60115, Indonesia; 3 Department of Mathematics, Faculty of Science and Technology, Airlangga University, Surabaya, 60115, Indonesia; 4 Department of Applied Mechanics and Design, Faculty of Mechanical Engineering, Universiti Teknologi Malaysia, Johor Bahru, 81310, Malaysia

**Keywords:** Blue-laser, methicillin-resistant *Staphylococcus aureus*, ozone therapy, red-laser

## Abstract

**Background and Aim:**

According to 2013 data from the Ministry of Health of the Republic of Indonesia, there were 8.2% more wounds than typical in Indonesia; 25.4% were open wounds, 70.9% were abrasions and bruises, and 23.2% were lacerations. A wound is defined as damage or loss of body tissue. This study aimed to determine the effectiveness of wound healing using red-laser therapy (650 nm, 3.5 J/cm^2^), blue-laser therapy (405 nm, 3.5 J/cm^2^), ozone therapy, red-laser therapy (650 nm, 3.5 J/cm^2^) with ozone, and blue-laser therapy (405 nm, 3.5 J/cm^2^) with ozone.

**Materials and Methods:**

One hundred and twelve mice were given incision wounds and infected with methicillin-resistant *Staphylococcus aureus* (MRSA). The study used a factorial design with two factors: The type of therapy (n = 7) and irradiation time (days 1, 2, 4, and 6). The mice were divided into seven therapy groups: Control group with NaCl, control with Sofra-tulle^®^ treatment, red-laser therapy (650 nm, 3.5 J/cm^2^), blue-laser therapy (405 nm, 3.5 J/cm^2^), ozone therapy, red-laser therapy (650 nm, 3.5 J/cm^2^) with ozone, and blue-laser therapy (405 nm, 3.5 J/cm^2^) with ozone. This therapy was performed using irradiation perpendicular to the wound area. The photosensitizer used was curcumin 10 mg/mL, which was applied to the wound area before exposure to a laser and ozone. The ozone concentration was 0.011 mg/L with a flow time of 80 s. The test parameters were the number of collagens, bacterial colonies, lymphocytes, monocytes, and wound length measurement to determine their acceleration effects on wound healing. Data were analyzed by a two-way (factorial) analysis of variance test.

**Results:**

Acceleration of wound healing was significantly different between treatments with a laser or a laser-ozone combination and treatment using 95% sodium chloride (NaCl) and Sofra-tulle^®^. On day 6, the blue-laser with ozone treatment group had efficiently increased the number of bacteria and reduced the wound length, and the red-laser treatment with ozone increased the amount of collagen. In addition, the red-laser also reduced the number of lymphocytes and monocytes, which can have an impact on accelerating wound healing. Blue-laser therapy was very effective for increasing the number of epithelia.

**Conclusion:**

The blue- and red-laser combined with ozone treatments effectively accelerated the healing of incisional wounds infected with MRSA bacteria.

## Introduction

The World Health Organization estimates that injuries claim the lives of >14,000 people every day. Self-harm or harming others, car accidents, drowning, falls, poisoning, and burns are some causes of injury [1]. According to the Indonesian Ministry of Health (2018), the prevalence of patients with wounds compared with the total number of patients in Indonesia was 8.2%, which included abrasions or bruises caused by rubbing the skin against rough surfaces (70.9%), cuts caused by sharp objects (25.4%), and lacerations caused by blunt objects (23.2%). A wound is defined as damage to or loss of body tissue, which can include side effects of medical care. Depending on the cause, wounds can take many shapes because of incisions from sharp objects [2]. Incisions are wounds caused by sharp objects, such as intentional or unintentional cuts during surgery. Wound characteristics include open injuries, pain, and length dimensions longer than the depth dimension. Physiologically, wound healing consists of three phases: Inflammation, proliferation, and remodeling or maturation [3]. Bacterial contamination that might result in infection is one of the barriers to wound healing [4]. Methicillin-resistant *Staphylococcus aureus* (MRSA) bacteria can infect numerous body parts.

Wounds infected with bacteria are generally treated with antibiotics. Long-term use of antibiotics or improper dosage to treat bacterial infections can cause bacteria to become resistant, such as MRSA. These infected wounds are difficult to heal. Therefore, alternative treatments are needed to overcome this problem. Photo biomodulation is a viable alternative therapy [5] that uses low-power laser light to hasten wound healing. The energy transmission in photo biomodulation does not produce harmful heat. Photo biomodulation therapy is thus relatively safer than other laser therapies. By increasing reactive oxygen species (ROS) and reducing reactive nitrogen species, lasers cause a shift in the cell’s total redox potential in favor of increased oxidation [6]. Reactive oxygen species function as a second messenger by stimulating neutrophils and monocytes, stabilizing transcription, and encouraging neutrophil monocyte attachment to the extracellular and vascular matrix, influencing adhesion molecule expression. The proliferation of fibroblasts, keratinocytes, endothelial cells, and lymphocytes is increased by laser therapy. Activating signaling pathways and elevating transcription factors, which ultimately increase growth factors, follow mitochondrial photo stimulation as the proliferation mechanism. Acute and chronic wound healing can also be aided by lasers by increasing neovascularization, enhancing angiogenesis, and stimulating collagen synthesis [7]. The antimicrobial photodynamic therapy (aPDT) effect is a non-thermal process involving chromophore changes through photophysical and photochemical processes at various biological scales to produce ROS that will inactivate pathogenic microorganisms and their endotoxins, it is another laser effect in addition to the photo biomodulation effect [8].

Porphyrin compounds, which function as light-sensitive photosensitizer molecules, naturally occur in some bacteria. By administering photosensitizers, such as turmeric, exogenously, aPDT effects can be improved (*Curcuma longa*) [9]. Curcumin has pharmacological effects against cancer, inflammation, free radicals, and germs. As part of its mechanism of action, PDT breaks down Gram-positive membranes, allowing curcumin compounds to interact directly with proteins in the canal and phospholipids in the cell wall. After the contact occurs, all ions enter the cell, causing cell lysis [10]. Bacteria are photo-inactivated when they are exposed to light and certain photosensitizers. This stops cell metabolism activation caused by ROS damage to the cytoplasmic membrane [11].

Ozone an unstable form of oxygen that is a potent oxidant with a pungent smell. Even compared with chlorine, ozone kills bacteria 3250 times more quickly than other disinfectants, so it can serve as a disinfectant. Ozone is also a gas that can be converted into oxygen atoms (O*) and oxygen gas molecules (O_2_) by absorbing ultraviolet light in a wavelength range of 240–340 nm. Ozone is added to wound therapy to enhance its capacity to kill infection-causing bacteria, strengthen tissue and blood circulation, hasten tissue epithelization, and promote cell regeneration [12]. By oxidizing H_2_O_2_ through the Fenton reaction, ozone creates the oxidant molecules H_2_O_2_ and free radicals, both of which boost immunity. Ozone can propagate to the cytoplasm even though it cannot enter the tissue [13]. The previous studies have shown that exposure to visible light photons and ozone can minimize bacterial biofilms [14, 15], similar to infrared light, which shows its ability to regenerate cells in patients with diabetes [16, 17]. In a mouse model with a full-thickness excision wound infected with MRSA germs, the previous studies utilizing RLP068/Cl in a gel formulation revealed a decrease in the number of bacteria in the lesion. In addition, RLP068/Cl can hasten wound healing [18]. A subsequent study found that photodynamic treatment effectively promoted wound healing in white mice with MRSA infections [19]. Other studies have shown that by decreasing wound contraction and angiogenesis, two crucial components of tissue integrity, non-steroidal anti-inflammatory drugs (NSAIDs) employed in this animal wound-healing model have been shown to somewhat impair the healing process [20]. Additional research has shown that NSAIDs may reduce oxidative stress and their anti-inflammatory benefits [21].

This study aimed to compare the efficacies of laser photodynamic therapy, the photosensitizer curcumin, and ozone therapy for the treatment of MRSA infections in incision wounds.

## Materials and Methods

### Ethical approval

The study was approved by Dental Medicine Health Research Ethical Clearance Commission of Airlangga University (approval no. 034/HRECC.FODM/I/202).

### Study period and location

The research was conducted from May to June 2022. Animal treatment, euthanasia, and surgery were performed at the Experimental Animal House, Faculty of Veterinary Medicine, Universitas Airlangga. Preparation and testing of histopathology, lymphocytes, monocytes, bacterial colonies, and wound length measurements were carried out at the Faculty of Veterinary Medicine Laboratory, Universitas Airlangga.

### Materials

Male BALB/c strain mice weighing 25 and 35 g were used as the test subjects. These mice had healthy physical characteristics, such as clear eyes, shiny leg hair, energetic movements, and excellent faces. A total of 112 mice were included and there were 16 mice per treatment group. The mice were separated into seven groups: The control with sodium chloride (NaCl) (K−), the control with Sofra-tulle^®^ (PT UBC Medical, Indonesia) treatment (K+), red-laser therapy (P1), blue-laser therapy (P2), ozone therapy (P3), red-laser therapy with ozone (P4), and blue-laser therapy with ozone (P5). A straightforward random sample technique was used to perform the sampling.

### Adaptation and maintenance procedures for experimental animals

The first stage of experimental animal care (acclimatization) is the adaptation of mice to their surroundings. The objectives of adapting experimental animals were to achieve the appropriate body weight, good general health, and environmental transformation. Because the mice were put in cages, this acclimatization phase lasted 7 days. A plastic cage covered in gauze was used as the animal enclosure for all mice. The room conditions and animal cages were ventilated and exposed to a 12-h light-12-h dark cycle. Food was provided every morning, midday, and night by putting it in a tiny container accessible to the mice. Water that had been heated previously was provided in a 300 mL bottle for ad libitum drinking.

### Anesthesia technique

This study used an anesthetic technique before creating an incision wound. The anesthesia comprised ether and carbon dioxide. The drug was placed on the bottom of a desiccator, the animal was inserted, and the container was closed. When the animal lost consciousness, it was able to be dissected while in the desiccator. Subsequent addition of ether in a cotton swab could be performed by placing the swab over the animal’s mouth and nose.

### Creation of an incision wound

The back hair of the mice had been shaved to provide a bare-skin surface. The backs of the mice were cleansed with 70% alcohol and cut on the right side with a mess hand vat. Measuring from the subcutis region, the wound was 2.5 cm long. Six groups of injured animals received treatment every other day for a week.

### Treatment

After the incision, the animals were treated with a 650 nm diode laser and a 405 nm blue-laser at the same dose. The therapy was performed by irradiating perpendicular to the wound area. This study used a photosensitizer of 10 mg/mL of curcumin, which was applied to the wound area before exposure to the laser and ozone [14, 15]. The ozone concentration given was 0.011 mg/L with a flow time of 80s [9]. The research samples were divided into seven therapy groups: The control with NaCl (K−), control and Sofra-tulle^®^ treatment control (K+), red-laser therapy^@^ 3.5 J/cm^2^ (P1), blue-laser therapy^@^ 3.5 J/cm^2^ (P2), ozone therapy (P3), red-laser therapy^@^ 3.5 J/cm^2^ with ozone (P4), and blue-laser therapy^@^ 3.5 J/cm^2^ with ozone (P5). The study used a factorial design with two factors: The type of therapy (K−, K+, P1, P2, P3, P4, and P5) and irradiation time (days 1, 2, 4, and 6), so there were 28 separate experiments with three replications.

### Procedures for euthanasia of experimental animals and collection of preparations

The mice used in the experiments were prepared and sacrificed 24 h after each group received their treatment. The mice were first put to death by cervical dislocation. By exerting pressure at the base of the skull, the euthanasia technique seeks to separate the head and brain from the spinal cord. The spinal cord controls heartbeat, stops breathing, and inhibits blood flow, all of which can result in death. Surgery can be performed if the mice do not respond. A scalpel can be used to cut and remove the mandibular tissue, which can then be placed in a urine pot with a 10% neutral formalin buffer solution. The solution prevents tissue stiffening or decay after the preparations have been obtained and can boost tissue affinity during immunohistochemistry testing. Mandibular preparations were kept only in storage for a maximum of 3 days.

### Procedures for making and examining histopathology preparations

A 10% buffered neutral formalin solution was used to fix tissue slices, followed by alcohol dehydration, \ Xylol for purification, and paraffin for molding or paraffinization. Microtome were used to cut or sliced the frozen paraffin blocks to a thickness of 5 µm. After drying, the preparation was placed on a glass surface and allowed to dry overnight at 60°C in an incubator.

### Statistical analysis

To determine the effect of each treatment, the research data for each parameter and each day of observation were subjected to two-way (factorial) analysis of variance statistical testing using IBM Statistical package for the social sciences (SPSS) 21.0 software (IBM SPSS Statistics for Windows, IBM Corp., Armonk, NY), followed by Tukey’s *post hoc* test (p<0.05).

## Results

### Effect of treatment on the epithelium

**Figure-1:**
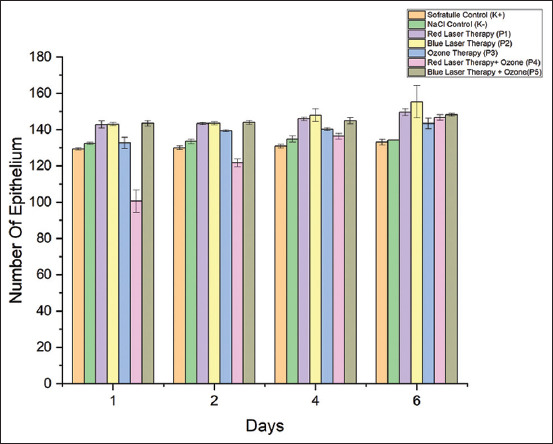
Comparison of epithelial counts from day 1 to day 6 in the control and treatment groups.

Table-1 shows a significant difference in the number of epithelia between treatments. *Post hoc* analysis showed that on day 6, mice treated with blue-laser therapy had the ideal number of epithelial layers, which was not substantially different from the number of layers in the mice treated with red-laser therapy and blue-laser therapy with ozone. Further evidence of the relationship between treatment and day is shown in Figure-1. It was evident that there was more epithelium on day 6 than on days 1, 2, and 4.

**Table-1:** Results of a two-way ANOVA factorial test on the number of epitheliums in the control and treatment groups.

Treatment	Epithelial cell count				Conclusion

	Day 1	Day 2	Day 4	Day 6	
Sofra-tulle control (K+)	129.33^b,c^ ± 0.58	129.89^c^ ± 1.02	130.83^c^ ± 1.04	133.11^c,d^ ± 1.65	p = 0.00 (there is a significant difference)
NaCl control (K−)	132.33^c,d^ ± 0.58	133.50^c,d^ ± 1.32	134.80^c,d,e^ ± 1.71	134.17^c,d^ ± 0.07	
Red-laser therapy (P1)	142.77^e,f,g,h,i^ ± 2.04	143.33^f,g,h,i^ ± 0.58	145.90^g,h,i^ ± 0.85	149.60^h,i,j^ ± 1.83	
Blue-laser therapy (P2)	143.00^e,f,g,h,i^ ± 1.00	143.50^f,g,h,i^ ± 0.87	148.00^h,i,j^ ± 3.46	155.33^j^ ± 8.95	
Ozone therapy (P3)	132.67^c,d^ ± 3.06	139.33^d,e,f,g^ ± 0.58	140.17^d,e,f,g,^ ^h^ ± 0.76	143.33^f,g,h,i^ ± 2.89	
Red-laser therapy+ozone (P4)	100.57^a^ ± 6.17	121.67^b^ ± 2.08	136.33^c,d,e,f^ ± 1.53	146.67^g,h,i^ ± 1.53	
Blue-laser therapy+ozone (P5)	143.50^f,g,h,i^ ± 1.51	144.00^f,g,h,i^ ± 1.00	144.87^g,h,i^ ± 1.63	148.20^h,i,j^ ± 0.72	

ANOVA=Analysis of variance, Significant differences are denoted by different letters within the same column (p < 0.05)

### Effect of treatment on collagen

**Figure-2:**
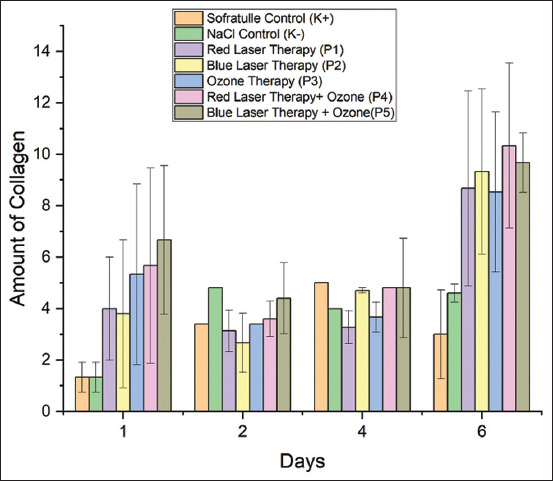
Comparison of the amount of collagen in the control and treatment groups from day 1 to day 2.

The effect of the wound-healing process on collagen was evaluated. Figure-2 shows that, on average, collagen production rises daily. There was a significant difference in collagen levels between treatments (p = 0.00) (Table-2). On day 6, the mice treated with red-laser therapy combined with ozone showed the highest level of collagen, which was not substantially different from the collagen level after blue-laser therapy with and without ozone, according to the findings of the *post hoc* test. Furthermore, time and treatment were related to one another; significantly more collagen was present on day 6 than on days 1, 2, and 4.

**Table-2:** Results of a two-way ANOVA factorial test on the amount of collagen in control and treatment groups.

Treatment	Amount of Collagen				Conclusion

	Day 1	Day 2	Day 4	Day 6	
Sofra-tulle control (K+)	1.33^a^ ± 0.58	3.40^a,b,c^ ± 0.00	5.00^a,b,c,d,e^ ± 0.00	3.00^a,b^ ± 1.73	p = 0.00 (there is a significant difference)
NaCl control (K−)	1.33^a^ ± 0.58	4.80^a,b,c,d,e^ ± 0.00	4.00^a,b,c,d^ ± 0.00	4.60^a,b,c,d,e^ ± 0.35	
Red-laser therapy (P1)	4.00^a,b,c,d^ ± 2.00	3.13^a,b,c^ ± 0.81	3.27^a,b,c^ ± 0.64	8.67^b,c,d,e^ ± 3.79	
Blue-laser therapy (P2)	3.80^a,b,c,d^ ± 2.88	2.67^a,b^ ± 1.15	4.70^a,b,c,d,e^ ± 0.10	9.33^c,d,e^ ± 3.21	
Ozone therapy (P3)	5.33^a,b,c,d,e^ ± 3.51	3.40^a,b,c^ ± 0.00	3.67^a,b,c,d^ ± 0.58	8.53^b,c,d,e^ ± 3.11	
Red-laser therapy+ozone (P4)	5.67^a,b,c,d,e^ ± 3.79	3.60^a,b,c,d^ ± 0.69	4.80^a,b,c,d,e^ ± 0.00	10.33^e^ ± 3.21	
Blue-laser therapy+ozone (P5)	6.67^a,b,c,d,e^ ± 2.89	4.40^a,b,c,d,e^ ± 1.39	4.80^a,b,c,d,e^ ± 1.93	9.67^d,e^ ± 1.15	

ANOVA=Analysis of variance, Significant differences are denoted by different letters within the same column (p < 0.05)

### Effect of treatment on the number of bacterial colonies

**Figure-3:**
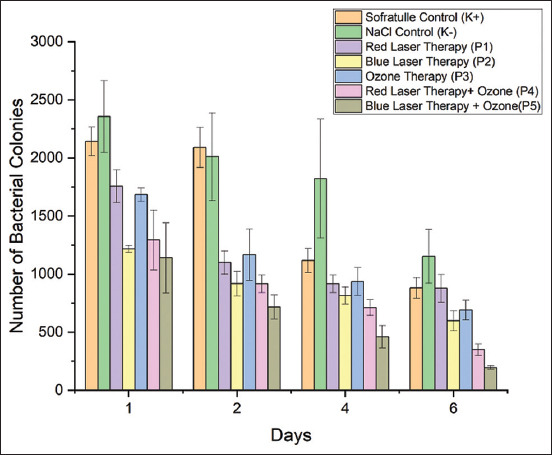
Comparison of bacterial colony counts on days 1–6 in the control and treatment groups.

To ascertain the growth of bacterial colonies in the cut wounds of the mice, the number of bacterial colonies was counted. Figure-3 demonstrates that fewer bacterial colonies were often present from day 1 to day 6. There was a significant difference in the number of bacterial colonies between treatments (p = 0.00) (Table-3). The ideal number of bacterial colonies was reduced in the mice receiving blue-laser therapy with ozone on day 6, which was not statistically different from the numbers of bacterial colonies in the mice receiving red-laser therapy with ozone, ozone therapy, and blue-laser therapy (p = 0.00, *post hoc* test). There was a relationship between treatment and the day post-treatment; the bacterial colony reduction was greater on day 6 than on days 1, 2, and 4 (p = 0.00, *post ho*c test).

**Table-3:** Factorial two-way ANOVA test results on the number of bacterial colonies with control and treatment groups.

Treatment	Number of bacterial colonies				Conclusion

	Day 1	Day 2	Day 4	Day 6	
Sofra-tulle control (K+)	2141.67^i,j^ ± 123.32	2090.00^i,j^ ± 173.49	1116.67^c,d,e,f^ ± 104.08	881.67^b,c,d,e^ ± 88.08	p = 0.00 (there is a significant difference)
NaCl control (K−)	2356.00^j^ ± 310.17	2010.67^i,^ ^j^ ± 377.64	1821.67^h,i,^ ^j^ ± 511.28	1152.67^c,d,e,f^ ± 232.64	
Red-laser therapy (P1)	1756.67^g,h,i^ ± 140.12	1100.00^c,d,e,f^ ± 100.00	916.67^b,c,d,e^ ± 76.38	878.67^b,c,d,e^ ± 119.44	
Blue-laser therapy (P2)	1216.67^d,e,f,g^ ± 28.87	918.33^b,c,d,e^ ± 106.11	815.33^b,c,d,e^ ± 74.20	597.67^a,b,c^ ± 84.65	
Ozone therapy (P3)	1683.33^f,g,h,i^ ± 57.74	1166.67^c,d,e,f,g^ ± 221.89	935.67^b,c,d,e^ ± 119.82	689.33^a,b,c,d^ ± 84.51	
Red-laser therapy+ozone (P4)	1294.00^e,f,g,h^ ± 257.89	916.67^b,c,d,e^ ± 76.38	711.67^a,b,c,d,e^ ± 68.25	350.00^a,b^ ± 50.00	
Blue-laser therapy+ozone (P5)	1140.00^c,d,e,f^ ± 300.50	716.67^a,b,c,d,e^ ± 104.08	460.00^a,b^ ± 96.44	196.00^a^ ± 14.42	

ANOVA=Analysis of variance, Significant differences are denoted by different letters within the same column (p < 0.05)

### Effect of treatment on lymphocyte cell count

**Figure-4:**
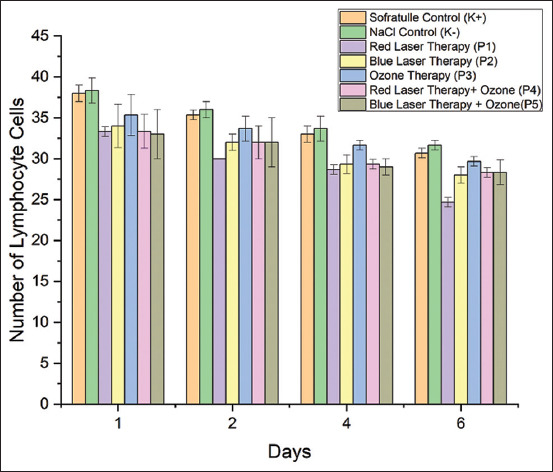
Comparison of lymphocyte cell counts of the control and treatment groups on days 1–6.

To evaluate the process of wound healing, lymphocyte cell counts were measured. Figure-4 shows that, on average, fewer lymphocyte cells were present from days 1 to 6. There was a significant difference in the number of lymphocytes between treatments (p = 0.00) (Table-4). On day 6, optimal reduction in the number of lymphocyte cells was observed in mice treated with red-laser therapy. Furthermore, time and treatment were related to one another. The lymphocyte cell count reduction was greater on day 6 than on days 1, 2, and 4.

**Table-4:** Factorial two-way ANOVA test results on lymphocyte cell counts with control and treatment groups.

Treatment	Lymphocyte Cell Count				Conclusion

	Day 1	Day 2	Day 4	Day 6	
Sofra-tulle control (K+)	38.00^h,i^ ± 1.00	35.33^g,h,i^ ± 0.58	33.00^c,d,e,f,g^ ± 1.00	30.67^b,c,d,e,f^ ± 0.58	p = 0.00 (there is a significant difference)
NaCl control (K−)	38.33^i^ ± 1.53	36.00^g,h,i^ ± 1.00	33.67^e,f,g,h^ ± 1.53	31.67^b,c,d,e,f,g^ ± 0.58	
Red-laser therapy (P1)	33.33^d,e,f,g^ ± 0.58	30.00^b,c,d,e,f^ ± 0.00	28.67^a,b,c^ ± 0.58	24.67^a^ ± 0.58	
Blue-laser therapy (P2)	34.00^f,g,h,i^ ± 2.65	32.00^b,c,d,e,f,g^ ± 1.00	29.33^b,c,d,e^ ± 1.15	28.00^a,b^ ± 1.00	
Ozone therapy (P3)	35.33^g,h,i^ ± 2.52	33.67^e,f,g,h^ ± 1.53	31.67^b,c,d,e,f,g^ ± 0.58	29.67^b,c,d,e,f^ ± 0.58	
Red-laser therapy+ozone (P4)	33.33^d,e,f,g^ ± 2.08	32.00^b,c,d,e,f,g^ ± 2.00	29.33^b,c,d,e^ ± 0.58	28.33^a,b^ ± 0.58	
Blue-laser therapy+ozone (P5)	33.00^c,d,e,f,g^ ± 3.00	32.00^b,c,d,e,f,g^ ± 3.00	29.00^a,b,c,d^ ± 1.00	28.33^a,b^ ± 1.53	

ANOVA=Analysis of variance, Significant differences are denoted by different letters within the same column (p < 0.05)

### Effect of treatment on monocyte cell count

**Figure-5:**
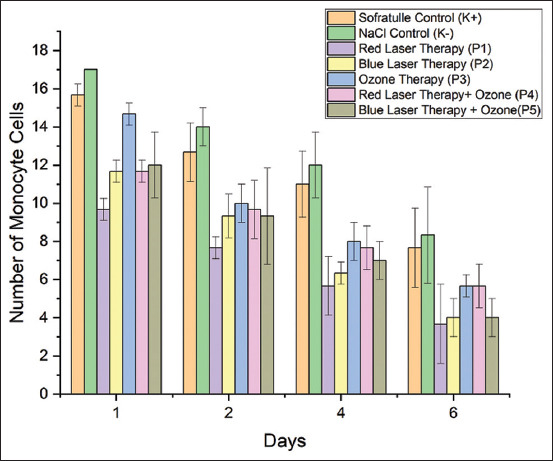
Monocyte cell counts in the control and treatment groups from day 1 to day 6.

The wound-healing process was determined by measuring the number of monocyte cells. Figure-5 shows that, on average, fewer monocyte cells were present from day 1 to day 6. There was a significant difference in the number of monocyte cells between treatments (p = 0.00) (Table-5). The greatest reduction in the number of monocyte cells was found in the mice treated with blue-laser therapy and blue-laser therapy combined with ozone on day 6. Furthermore, time and treatment were related to one another. The reduction in monocyte cell counts was greater on day 6 than on days 1, 2, and 4.

**Table-5:** Results of a two-way ANOVA factorial test on monocyte cell counts with control and treatment groups.

Treatment	Monocyte Cell Count				Conclusion

	Day 1	Day 2	Day 4	Day 6	
Sofra-tulle control (K+)	15.67^k,l^ ± 0.58	12.67^h,i,j,k^ ± 1.53	11.00^e,f,g,h,i,j^ ± 1.73	7.67^a,b,c,d,e,f^ ± 2.08	p = 0.00 (there is a significant difference)
NaCl control (K−)	17.00^l^ ± 0.00	14.00^i,j,k,l^ ± 1.00	12.00^g,h,i,j,k^ ± 1.73	8.33^c,d,e,f,g^ ± 2.52	
Red-laser therapy (P1)	9.67^c,d,e,f,g,h^ ± 0.58	7.67^a,b,c,d,e,^ ^f^ ± 0.58	5.67^a,b,c^ ± 1.53	3.67^a^ ± 2.08	
Blue-laser therapy (P2)	11.67^f,g,h,i,j,k^ ± 0.58	9.33^c,d,e,f,g,h^ ± 1.15	6.33^a,b,c,d^ ± 0.58	4.00^a,b^ ± 1.00	
Ozone therapy (P3)	14.67^j,^ ^k,^ ^l^ ± 0.58	10.00^d,e,f,g,h,i^ ± 1.00	8.00^b,c,d,e,f,g^ ± 1.00	5.67^a,b,c^ ± 0.58	
Red-laser therapy+ozone (P4)	11.67^f,g,h,i,j,k^ ± 0.58	9.67^c,d,e,f,g,h^ ± 1.53	7.67^a,b,c,d,e,f^ ± 1.15	5.67^a,b,c^ ± 1.15	
Blue-laser therapy+ozone (P5)	12.00^g,h,i,^ ^j^.^k^ ± 1.73	9.33^c,d,e,f,g,h^ ± 2.52	7.00^a,b,c,d,e^ ± 1.00	4.00^a,b^ ± 1.00	

ANOVA=Analysis of variance, Significant differences are denoted by different letters within the same column (p < 0.05)

### Effect of treatment on macroscopic features

**Figure-6:**
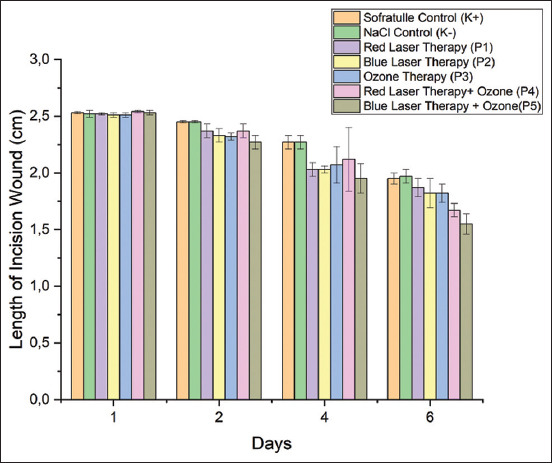
Comparison of wound length in the control and treatment groups from day 1 to day.

Macroscopic imaging was performed to measure the length of the mouse incision wound. The incision wound decreased from day 1 to day 6 (Figure-6). There was a significant association between treatment and incision wound length (p = 0.00) (Table-6). On day 6, there was a significant decrease in the incision-woundlength in the mice treated with blue-laser combined with ozone therapy, which was not substantially different from those treated with red-laser therapy combined with ozone and blue-laser and ozone therapy. The incision length reduction was greater on day 6 than on days 1, 2, and 4, indicating a significant association between the incision length reduction and the post-treatment day (p=0.00, *post ho*c test).

**Table-6:** Results of a factorial two-way ANOVA test on the length of incision wounds in mice with control and treatment groups.

Treatment	Length of incision wound				Conclusion

	Day 1	Day 2	Day 4	Day 6	
Sofra-tulle control (K+)	2.53^i^ ± 0.01	2.45^h,i^ ± 0.01	2.27^e,f,g,h^ ± 0.06	1.95^c,d^ ± 0.05	p = 0.00 (there is a significant difference)
NaCl control (K−)	2.52^h,i^ ± 0.03	2.45^h,i^ ± 0.01	2.27^e,f,g,h^ ± 0.06	1.97^c,d^ ± 0.06	
Red-laser therapy (P1)	2.52^h,i^ ± 0.01	2.37^g,h,i^ ± 0.06	2.03^c,d,e^ ± 0.06	1.87^b,c,d^ ± 0.08	
Blue-laser therapy (P2)	2.51^h,i^ ± 0.02	2.33^f,g,h,i^ ± 0.06	2.03^c,d,e^ ± 0.03	1.82^a,b,c^ ± 0.13	
Ozone therapy (P3)	2.51^h,i^ ± 0.02	2.32^f,g,h,i^ ± 0.03	2.07^c,d,e,f^ ± 0.16	1.82^a,b,c^ ± 0.08	
Red-laser therapy+ozone (P4)	2.54^i^ ± 0.01	2.37^g,h,i^ ± 0.06	2.12^d,e,f,g^ ± 0.28	1.67^a,b^ ± 0.06	
Blue-laser therapy+ozone (P5)	2.53^h,i^ ± 0.02	2.27^e,f,g,h^ ± 0.06	1.95^c,d^ ± 0.13	1.55^a^ ± 0.09	

ANOVA=Analysis of variance, Significant differences are denoted by different letters within the same column (p < 0.05)

## Discussion

The therapeutic use of non-invasive and low-power light energy is known as PDT or sometimes phototherapy, photo radiation therapy, or photochemotherapy. Photodynamic therapy uses three non-toxic substances: Oxygen, a non-toxic photosensitizer, and harmless visible light. Photosensitizers absorb light energy at specific wavelengths, creating a range of radical products that destroy pathogenic microorganisms. Curcumin is used as a photosensitizer in conjunction with ozone therapy. In this study, wounds were created by opening the backs of mice and injecting them with MRSA until pus formed as a sign of microbial infection. Simultaneous random therapy also was administered to each group. Until day 6, treatment was performed every day with 24 h intervals. After each therapy was administered to each mouse for 24 h, tissue samples were taken. An increase in pro-inflammatory cells, specifically fibroblast cells, the development of new blood vessels, and an increase in lymphocyte cells that speed wound healing have all been observed in the previous research using red-lasers on post-tooth extraction wounds [7]. The results of immunohistochemical tests supported the effects of photobiomodulation in this study, which showed an increase in the expression of Col-1α protein that has a role in collagen formation as a form of new tissue after tissue damage occurs, as well as a decrease in interleukin (IL)-1β expression levels.

In the previous study, it was found that red-laser light exposure to wounds was able to accelerate wound healing [9]. This was shown by a significant increase in pro-inflammatory cells (specifically fibroblast cells), the formation of new blood vessels, and an increase in lymphocyte cells. Immunohistochemical test results have shown an increase in the expression of Col-1α protein, which has a role in collagen formation as a form of new tissue after tissue damage occurs, and a decrease in IL-1β expression.

The inflammatory, proliferation, and maturation phases comprise the burn-wound healing process. Days 0 through 5 are spent in the inflammatory phase, and days 3 through 14 are spent in the proliferation phase. Accordingly, day 4 may occur in the proliferative or inflammatory phase depending on the animal’s health. Consequently, epithelium growth starts on day 4. It slows down on day 6 because sodium and potassium levels are absorbed by cells at the wound edge so that granulation growth in epithelial tissue is inhibited. Fibroblasts create vast amounts of collagen, a glycoprotein that helps create strength in scar tissue, which accounts for the increased quantity of fibroblasts in the wound area. The amount of epithelium was considerably higher in the blue-laser therapy group than in the other groups (Tukey’s *post hoc* test results).

Compared with the collagen amounts in the other treatment groups, the collagen was significantly higher in the ozone-laser combination and red-laser therapy groups. Collagen’s capacity to bind fibronectin can aid platelet aggregation [22]. Although the precise mechanism of collagen interaction is unknown, conclusive evidence suggests that the exchange of collagen and platelets, also known as homeostasis, is the initial stage of the healing process, followed by vasoconstriction and vasodilation. Vasodilation makes the non-traumatized area more permeable, so hormones, plasma proteins, electrolytes, antibodies, fluids, and polymorphonuclear (PMN) leukocytes can move through [23]. Vasodilatation and cleaning of the wound region follow vasoconstriction and occur in that order. Polymorphonuclear leukocytes and macrophages rapidly accumulate at the trauma site. Monocytes can be drawn to collagen through chemotaxis. Similar to macrophages, monocytes phagocytose microorganisms in the vicinity of the wound and remove waste.

The endotoxins produced can penetrate easily due to skin and tissue integrity loss. This causes the patient’s immune system to overreact, possibly causing the immune system to malfunction [24]. On day 6, the body will also undergo a phase of resistance to germs by leukocytes when these bacteria start interfering with the immune system, which can lead to a decrease in germ colonies.

The number of lymphocytes increases during an inflammatory response because they travel to the location of the wound on day 1, reach a peak between days 3 and 6, and then start to decline on day 7. Inflammation requires both humoral and cellular responses from lymphocytes. A lymphocyte is one of the first cells to arrive at the wound site. They are activated and release lymphokines, including interferon, as part of their significant role in fighting infection by removing cellular matrix debris and foreign substances that attach to antigens. During phagocytosis, lymphokines help to stimulate and activate macrophages. Macrophages are responsible for phagocytizing maladapted cells and apoptotic PMN cells. Lymphocytes are activated by the cytokines IL-1 and tumor necrosis factor released by activated macrophages. To eliminate the triggering antigen agent, lymphocytes and macrophages constantly encourage one another, and fibroblasts have established a robust network [25]. In the present study, the Tukey *post hoc* test results of lymphocyte between the red-laser therapy group and the other groups. According to the idea that lymphocyte levels will increase during the proliferation phase because lymphocytes bind antigens and then become activated and release lymphokines, on day 6 lymphocyte levels in red-laser therapy were considerably decreased. During phagocytosis, lymphokines will play a part in stimulating and activating macrophages. Since monocytes now perform the role that lymphocytes initially did, a decrease in lymphocytes increases the likelihood that the inflammatory phase will last longer.

Leukocytes release hydrolytic enzymes that aid in the wound’s digestion of debris and germs. Many cellular effectors must infiltrate the wound region to combat the presence of foreign bodies on day 4, which is the inflammatory phase. Therefore, topical therapy is said to be effective if there is an increase in the number of cellular effectors, one of which is lymphocytes. During this stage, monocytes are involved in the phagocytosis of foreign objects. Therefore, if monocyte levels decrease, more foreign bodies will be phagocytosed, halting the progression of the inflammatory phase [26].

Monocyte counts were significantly higher in the red-laser therapy group than in the other groups (Tukey *post hoc* test). Macrophages participate in the proliferative and inflammatory stages and phagocytose more bacteria as they mature. Macrophages phagocytose the area of the wound and remove debris; their numbers increase during the inflammatory phase and decrease during the proliferative phase as the wound closes. Macrophages are generated from monocytes [27].

Within the first 24 h following a wound with a skinny layer, histopathological analysis shows white blood cells, in particular neutrophils, that appear at the wound edge and migrate toward the fibrin clot, causing the scab to appear to protect the wound from an inflammatory reaction or until infection due to bacterial microorganisms occurs. The wound length was significantly shorter in the blue-laser with ozone combination therapy group than in the other groups (Tukey *post hoc* test), which suggests that this treatment speeds up wound healing.

The study data showed that blue-laser therapy with ozone was highly effective in reducing the number of bacterial colonies and shortening the wound length. Monocyte and lymphocyte counts were significantly reduced by red-laser therapy. In addition, ted-laser therapy combined with ozone boosted collagen levels, and blue-laser therapy contributed to greater epithelial growth.

## Conclusion

The study results showed that NaCl and Sofra-tulle^®^ treatments, laser, and laser-ozone treatments significantly influenced the wound healing rate. On day 6, the blue-laser combined with the ozone therapy group successfully boosted the number of bacteria while shortening the length of the wound. Simultaneously, the amount of collagen increased following red-laser combined with ozone therapy. The red-laser also reduced the number of lymphocytes and monocytes, which hastened the wound-healing process. Blue-laser therapy also was very effective in promoting epithelial growth. Ozone-laser therapy hastened the recovery of incisional wounds contaminated with MRSA bacteria.

## Data Availability

The datasets used and/or analyzed during the study can be available from the corresponding author on a reasonable request.

## Authors’ Contributions

SDA: Conception of the study, data curation, methodology, analysis, project administration, supervision, validation, drafted and revised the manuscript. WIP, PADP, and DZIN: Conception of the study, data curation, methodology, investigation, validation, original draft preparation, and editing of the manuscript. SPAW: Conception of the study, methodology, investigation, analysis, supervision, validation, review, original draft preparation, and editing of the manuscript. AS: Conception of the study, methodology, analysis, supervision, validation, review, original draft preparation, and editing of the manuscript. All authors have read, reviewed, and approved the final manuscript.

## References

[1] WHO (2014). Injuries and Violence:The Facts.

[2] Martinengo L, Olsson M, Bajpai R, Soljak M, Upton Z, Schmidtchen A, Car J, Järbrink K (2019). Prevalence of chronic wounds in the general population:Systematic review and meta-analysis of observational studies. Ann. Epidemiol.

[3] De Oliveira Gonzalez A.C, Costa T.F, de Araújo Andrade Z, Medrado A.R.A.P (2016). Wound healing - A literature review. An. Bras. Dermatol.

[4] Tottoli E.M, Dorati R, Genta I, Chiesa E, Pisani S, Conti B (2020). Skin wound healing process and new emerging technologies for skin wound care and regeneration. Pharmaceutics.

[5] Hamblin M.R (2018). Mechanisms and mitochondrial redox signaling in photobiomodulation. Photochem. Photobiol.

[6] De Faria C.M.G, Costa C.S, Bagnato V.S (2021). Photobiomodulation effects on photodynamic therapy in HNSCC cell lines. J. Photochem. Photobiol. B.

[7] Astuti S.D, Sulistyo A, Setiawatie E.M, Khasanah M, Purnobasuki H, Arifianto D, Susilo Y, Alamsyah K.A, Suhariningsih, Syahrom A (2022). An *in-vivo* study of photobiomodulation using 403 nm and 649 nm diode lasers for molar tooth extraction wound healing in wistar rats. Odontology.

[8] Oruba Z, Łabuz P, Macyk W, Chomyszyn-Gajewska M (2015). Antimicrobial photodynamic therapy-A discovery originating from the pre-antibiotic era in a novel periodontal therapy. Photodiagnosis Photodyn. Ther.

[9] Astuti S.D, Mawaddah A, Nasution A, Mahmud A.F, Fitriyah N, Kusumawati I, Latief A, Puspita P.S, Suhariningsih (2020). Effectiveness of photodynamic inactivation with exogenous photosensitizer Curcuma longa extract activated by laser diode 403 nm on *Staphylococcus aureus*. J. Int. Dent. Med. Res.

[10] Dai T (2017). The antimicrobial effect of blue light:What are behind?. Virulence.

[11] Setiawatie E.M, Astuti S.D, Zaidan A.H (2016). An *in vitro* anti-microbial photodynamic therapy (aPDT) with blue LEDs to activate chlorophylls of alfalfa *Medicago sativa* L on *Aggregatibacter actinomycetemcomitans*. J. Int. Dent. Med. Res.

[12] Onyango A.N (2016). Endogenous generation of singlet oxygen and ozone in human and animal tissues:Mechanisms, biological significance, and influence of dietary components. Oxid. Med. Cell. Longev.

[13] Bocci V, Borrelli E, Travagli V, Zanardi I (2009). The ozone paradox:Ozone is a strong oxidant as well as a medical drug. Med. Res. Rev.

[14] Astuti S.D, Drantantiyas N.D.G, Putra A.O, Puspita P.S, Syahrom A, Suhariningsih S (2019). Photodynamic effectiveness of laser diode combined with ozone to reduce *Staphylococcus aureus* biofilm with exogenous chlorophyll of *Dracaena angustifolia* leaves. Biomed. Photonics.

[15] Puspita P.S, Astuti S.D, Nasution A.M.T, Pradhana A.A.S, Mawaddah A (2020). Photodynamic therapy with ozone aids to *Staphylococcus aureus* biofilm reduction. Indian Vet. J.

[16] Suhariningsih S, Astuti S.D, Husen S.A, Winarni D, Rahmawati D.A, Mukti A.T, Miftahussurur M (2020). The combined effect of magnetic and electric fields using on/off infrared light on the blood sugar level and the diameter of Langerhans islets of diabetic mice. Vet. World.

[17] Suhariningsih Dwi, W, Husen S.A, Firas K, Pramudita P.A, Astuti S.D (2020). The effect of electric field, magnetic field, and infrared ray combination to reduce HOMA-IR index and GLUT 4 in diabetic models of Mus musculus. Lasers Med. Sci.

[18] Simonetti O, Cirioni O, Orlando F, Alongi C, Lucarini G, Silvestri C, Zizzi A, Fantetti L, Roncucci G, Giacometti A, Offidani A, Provinciali M (2011). Effectiveness of antimicrobial photodynamic therapy with a single treatment of RLP068/Cl in an experimental model of *Staphylococcus aureus* wound infection. Br. J. Dermatol.

[19] Parani M, Lokhande G, Singh A, Gaharwar A.K (2016). Engineered nanomaterials for infection control and healing acute and chronic wounds. ACS Appl. Mater. Interfaces.

[20] Erpek S, Kilic N, Kozaci D, Dikicioglu E, Kavak T (2006). Effects of flunixin meglumine, diclofenac sodium and metamizole sodium on experimental wound healing in rats. Rev. Méd. Vét.

[21] Kılıç N, Kozacı L.D (2016). Effects of flunixine meglumine, diclofenac sodium, metamizol sodium and carprofen on oxidative stress in rats subjected to laparotomy. Sağlık Bilimleri Vet. Derg. Fırat Üniv.

[22] Hochstein A.O, Bhatia A.A (2023). Collagen:Its Role in Wound Healing. Podiatry Management.

[23] Mathew-Steiner S.S, Roy S, Sen C.K (2021). Collagen in wound healing. Bioengineering (Basel).

[24] Majtan J (2014). Honey:An immunomodulator in wound healing. Wound Repair Regen.

[25] Hofmann U, Frantz S (2015). Role of lymphocytes in myocardial injury, healing, and remodeling after myocardial infarction. Circ. Res.

[26] Thomsen K, Trøstrup H, Christophersen L, Lundquist R, Høiby N, Moser C (2016). The phagocytic fitness of leucopatches may impact the healing of chronic wounds. Clin. Exp. Immunol.

[27] Snyder R.J, Lantis J, Kirsner R.S, Shah V, Molyneaux M, Carter M.J (2016). Macrophages:A review of their role in wound healing and their therapeutic use. Wound Repair Regen.

